# Microbial Primer: Bacterial growth kinetics

**DOI:** 10.1099/mic.0.001428

**Published:** 2024-02-08

**Authors:** Lorena T. Fernández-Martínez, Arnaud Javelle, Paul A. Hoskisson

**Affiliations:** ^1^​ Institute of Infection, Immunity and Inflammation, University of Glasgow, Glasgow, G12 8TA, UK; ^2^​ Strathclyde Institute of Pharmacy and Biomedical Sciences, University of Strathclyde, 161 Cathedral Street, Glasgow, G4 0RE, UK

**Keywords:** bacterial growth, chemostat, growth curve

## Abstract

Growth of microorganisms and interpretation of growth data are core skills required by microbiologists. While science moves forward, it is of paramount importance that essential skills are not lost. The bacterial growth curve and the information that can gleaned from it is of great value to all of microbiology, whether this be a simple growth experiment, comparison of mutant strains or the establishment of conditions for a large-scale multi-omics experiment. Increasingly, the basics of plotting and interpreting growth curves and growth data are being overlooked. This primer article serves as a refresher for microbiologists on the fundamentals of microbial growth kinetics.

## Full-Text

‘The dream of every cell is to become two cells.’

**Francois Jacob**

Paraphrased by Jacques Monod in ‘Chance and Necessity’ 1970 (p. 20).

‘The study of the growth of bacterial cultures does not constitute a specialised subject or branch of research: it is the basic method of microbiology.’

**Jacques Monod**, 1949 *Annual Review of Microbiology*.

## Introduction

Understanding how a microbial cell functions is still not truly understood [1]. Experiments to achieve this goal begin with the cultivation of the organism of interest in a suitable growth medium – the art of microbiology. The growth of microorganisms and the interpretation of their kinetics is a core skill for microbiologists, one that is considered so straightforward that the basics are learnt and then promptly forgotten. Many scientific disciplines are driven by innovations in technology and techniques, often to the detriment of established and robust methodologies. The emergence of molecular biology as a major technique, driven by microbial research, led to basic skills in microbial physiology and metabolism becoming somewhat overlooked by new generations of scientists who gravitated to exciting technological innovations [2]. The molecular biology revolution enabled the birth of genomics, which subsequently led to the emergence of functional genomics (high-throughput mutagenesis, genome-scale reporters, etc.) and multi-omics methodology (transcriptomics, metabolomics and proteomics) as major tools for studying the biology of (micro)organisms. Whilst these new techniques are attractive to researchers, there has never been more of a need to focus on well-informed, basic microbial physiology to ensure that robust, high-quality material is generated for these analyses [1]. Poorly designed ‘omics experiments leads to the generation of poor-quality data, resulting in the old adage of ‘garbage in, garbage out’. As multi-omic, single cell ‘omics and high-resolution imaging techniques become increasingly accessible to researchers, there is an ever increasing need to highlight the skills in basic microbial physiology to ensure that the material for downstream study is generated with consideration given to the physiology of the organism.

The scientific study of microbial cultivations can be traced back to the mid-19th century, when it was recognized that yeasts were responsible for wine fermentations, followed by the pioneering work of Pasteur on aseptic methods and defining the growth conditions for microorganisms [3]. The development of the first defined media in the late 19th century followed by the introduction of shake flask cultures in the 1930s were perhaps the foundations of modern microbial physiology. The landmark monograph of Monod served as a driver of the golden age of microbial physiology in the 1950s, 1960s and 1970s, demonstrating that growth of microbial cultures could be described mathematically in terms of their growth-limiting substrates, growth yield and specific growth rate [3]. Further technical advances in the study of microbial physiology, such as the simultaneous invention of the chemostat by Monod in Paris and Novack and Szilard in Chicago [4], further enabled the development of the theory of microbial growth kinetics. Increasingly, researchers are overlooking these fundamental skills when performing experiments, which can hinder the comparison of cultures and verification of the data obtained. In recent years the erroneous reporting of linear-scale growth curves in publications, posters and presentations is occurring with increasing frequency. Growth and physiology have profound effects on the outputs from experiments and this Primer serves as a refresher of why good bacterial physiology matters to all microbiology experiments.

## The bacterial growth curve: a fundamental skill for microbiologists

Biological systems share several fundamental characteristics: order, the ability to sense and respond to external stimuli, regulation, substrate uptake, energy processing and growth (reproduction). Many, if not all, of these characteristics ultimately affect the rate of growth and reproduction of microbial systems. Thus, the monitoring of growth over time can reveal fundamental insight into the physiology of a biological system under a given set of conditions. It also allows quantitative comparison of strains and growth conditions, which is vitally important in inferring the effects of perturbations to the system on the growth of microorganisms (be that external chemical perturbations or studies of mutants). Indeed, the accurate recording of the specific growth rate of an organism is the best way to establish and validate the physiological state of a culture being studied [5]. The features of bacterial growth and the generality of findings by numerous bacterial physiologists have been fundamental to our understanding of not only growth, but also of non-growth states, response to perturbations, persistence and bacterial developmental processes. Many of the key findings of ‘early’ physiological studies have been shown to hold true across many prokaryotes during exponential growth and were summarized elegantly by Niedhardt [5]. Differential gene expression is dependent on environmental conditions, when cells are growing at a constant temperature and nutrients are in excess – each cell increases in size and volume by the same proportion in each time interval – so-called balanced growth. The specific growth rate of an organism coordinates with major physiological characteristics, such as cell size, chemical composition, etc., and when environmental conditions result in a change in specific growth rate, the patterns of macromolecular synthesis in response to that change are consistent, with concomitant alterations in RNA, protein and DNA synthesis [5]. These observations led to key discoveries in microbiology, for example that specific molecular mechanisms coordinate large sets of unlinked genes during growth and the elucidation of global metabolic control systems such as the stringent response and catabolite repression [5]. These examples provide the historical context for the development of integrationist approaches to studying microorganisms and these approaches lend themselves well to modern multi-omic studies – a truly integrative biology can only emerge from high-quality physiological approaches being linked with cutting edge technologies.

## The growth curve

The bacterial growth curve is a marvellous resource – the information contained within is invaluable, but often overlooked. The growth curve has discrete and recognizable phases that reflect distinct physiological states of the cells in culture: the lag phase as the organism adjusts to environmental conditions and adjusts its physiology to enable rapid growth; the exponential growth phase, where growth is constant and rapid – this phase is the most reproducible phase of growth and can allow direct comparisons between strains and conditions; and the stationary phase, where growth plateaus due to nutrient limitation, followed by the death phase, resulting from cell lysis due to severe limitation of nutrients. Quantifying microbial growth can be achieved in numerous ways, for example measuring the optical density of cell suspensions in a spectrophotometer, where the amount of light scatter is proportional to the concentration of cells in suspension. This works well for unicellular organisms, although caution is required when performing these analyses due to the non-linearity of spectrophotometers with high-density cultures. This is a problem that is particularly relevant when using 96-well plate readers to automate and measure growing cultures in parallel. To measure growth in aggregating organisms or filamentous species (bacteria and fungi), the determination of the dry weight of the cellular material in a known volume of culture is often used. This can be achieved through filtration and then drying cells to a constant weight. Other methods can also be used, such as packed cell volume, wet cell weight, total protein content and total viable cells via plate counting. The method of choice is often dictated by the nature of the medium used or the growth habit of the organism.

**Fig. 1.**
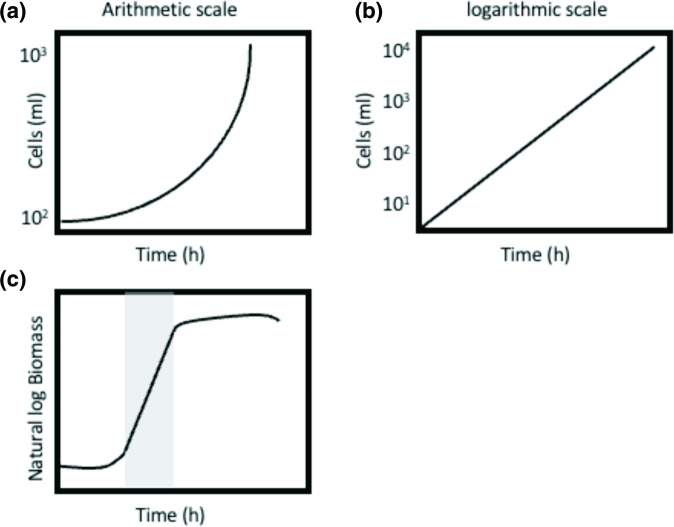
Plots illustrating the growth of microbial populations. (**a**) Plotting cell numbers against time on an arithmetic scale gives rise to a curve when growth is exponential. (**b**) When the same data are plotted on a semi-logarithmic scale the relationship is linear, and the slope of the line is equal to the specific growth rate, *µ*. (**c**). When considering the increase in biomass, rather than cell numbers, the natural logarithm of biomass plotted against time results in a straight line during the exponential phase of growth (grey), and again the slope of the line is equal to the specific growth rate, *µ* (h^−1^).

In microbial growth, where cells divide by binary fission, there is a proportional increase in all chemical components of the cell when nutrients are in excess (balanced growth) and when plotted on an arithmetic scale against time the data form a curve (Fig. 1a), indicating that growth is exponential (one cell becomes two, two become four and so on). Transforming these data to a semi-logarithmic plot of growth, in which the logarithm of cell numbers is plotted again time, reveals a linear relationship, equivalent to the equation of a straight line (*y*=*mx*+*C*; Fig. 1b). This is the exponential phase of growth, and the slope of this plot corresponds to the specific growth rate, *µ*. This is sometimes referred to as the growth rate constant, *k*, when dealing with cell numbers. The transformation of data in this manner enables the direct comparison of growth rates between cultures (wild-type strains vs mutants, strains on different carbon sources, the effects of exogenous additives, etc.). Plotting data in this manner also assists in reproducibility, as the growth rate constant in the exponential phase should be the same for strains grown under the same conditions.

Microbial growth can be expressed in the simplest of terms with the following equation:

(1) dN/dt=kN

where *N* is the number of cells, *t* is time and *k* is the first-order rate constant with unit of reciprocal hours (h^−1^).

Moreover, given that during balanced growth a microorganism behaves as an autocatalytic chemical reaction, the rate of growth is also proportional to the increase in cellular biomass (Fig. 1c). Given this, growth can then also be described by biomass (*X*) rather than cell numbers, where the rate of change in biomass can be described by:

(2) dX/dt=μX

where *X* is the concentration of biomass, *t* is time and *µ* is the specific growth rate with unit of reciprocal time (h^−1^).

Equation 2 can also be rearranged to estimate the specific growth rate (*µ*):

(3) μ=1/X.dX/dt

and equation 3 can be integrated to give:

(4) $$X_{t}=X_{0}e^{\mu t}$$

where *X*_*t*_ is the biomass concentration after time *t*, *X*_0_ is the initial biomass concentration, e is the base of the natural logarithm and *µ* is the specific growth rate, such that when taking the natural logarithm:

(5) $$ln\;X_{t}=ln\;X_{0}+\mu t$$

it provides an equation in the form of a straight line (*y*=*mx*+*c*). This straight line (a plot of the natural log of biomass concentration against time) gives a slope equal to *µ*, or:

(6) $$\mu =(ln\;X_{t}-ln\;X_{0})\;/\;t$$

The straight-line of these semi-logarithmic graphs displayed when the cells are in the exponential phase of growth is when nutrients are in excess and the organism is growing at a theoretical maximum specific growth rate, *µ*_max_ (Fig. 1c). The growth of microorganisms results in the consumption of nutrients and the excretion of waste and microbial products into the environment and these processes influence the growth of the culture. This results in the eventual cessation of growth due to the limitation of a substrate or the accumulation of an inhibitory product. This leads to the characteristic microbial growth curve we are all familiar with (Fig. 1c). The slope of the straight line of this semi-logarithmic plot is equal to the specific growth rate, *µ* (srey area in Fig. 1c).

## Measuring residual substrates and product formation can be informative

**Fig. 2.**
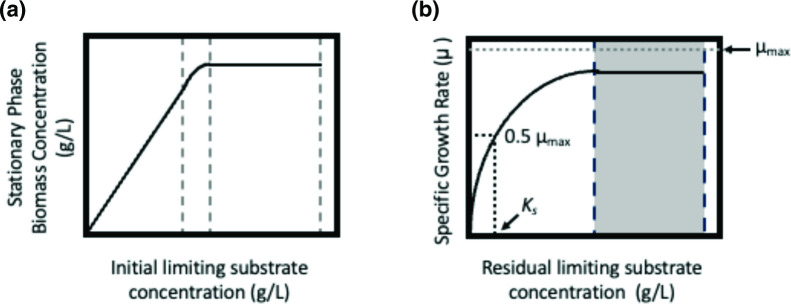
Plots illustrating the relationship between growth of microbial cultures and growth-limiting substrates. (**a**) The effect on biomass at stationary phase as a function of initial substrate concentration. (**b**) The effect of residual growth-limiting substrate on specific growth rate, *µ*. The region in grey is equivalent to the exponential phase of growth, essentially *µ*_max_. The estimation of *K*_s_ is indicated with the dotted line, where *s*=*K*_s_, *µ*=0.5*µ*_max_.

The limitation of growth due to substrate availability can be examined by measuring growth over an increasing concentration of the substrate and plotting the stationary phase biomass against initial substrate concentration (Fig. 2a). In the majority of laboratory experiments rich medium will be used (such as Lysogeny Broth or equivalent), but the use of minimal media (M9 or equivalent), with defined concentrations of nutrients, can allow the bacterial physiologist to learn huge amounts about their organism of choice and how it responds to environmental conditions. In simple experiments where a series of cultures are used to measure growth in response to increasing amounts of a substrate, the growth of the organism is proportional to the increasing substrate concentration, and it can be described by equation 7:

(7) $$X=Y(S_{i}-s)$$

where *X* is the concentration of biomass at stationary phase, *Y* is the yield (gram of biomass produced per gram of substrate), *S*_i_ is the initial substrate concentration and *s* is the residual substrate concentration. When the initial substrate concentration no longer continues to give a proportional increase in biomass (area within the dotted lines in Fig. 2a), this is probably due to the limitation of another substrate, or the accumulation of inhibitory product or waste. The efficiency of substrate conversion into a product (biomass in this example but can also be any other type of product) is the yield (*Y*) and this can be used to predict the amount of substrate required to produce a certain biomass concentration. This was first shown in the classic publication of Bauchop and Elsden [6], where the yield of ATP and biomass could be determined for a range of substrates. It should be noted that yield (*Y*) is not a constant and will vary according to the medium, growth rate, pH, etc.

More simply, the growth yield of a culture can be defined by the quotient:

(8) Y=ΔX /Δs

The increase of biomass (∆*X*) that results from the consumption of substrate (∆*s*) results in the yield, which can be an important way to express nutrient requirements in a quantitative manner. Monod [3] first showed that in bacterial cultures, under constant conditions, the growth yield (*Y*) is consistent and reproducible. Moreover, depletion of the limiting nutrient in a culture will result in a decrease in the growth rate and can be described by the relationship between the specific growth rate (*µ*) and the residual growth-limiting substrate concentration (Fig. 2b; [3]):

(9) $$\mu =\mu_{max}s\;/\;(K_{s}+s)$$

where *s* is the residual growth-limiting substrate concentration, *µ*_max_ is the maximum specific growth rate, and *K_s_* is the substrate utilization constant, which is numerically equal to the substrate concentration when *µ* is half the maximum specific growth rate (*µ*_max_), essentially a measure of substrate affinity. This equation will appear familiar to readers as it describes a single reaction rate (balanced growth) and its relationship to the concentration of a single substrate – the Michaelis–Menten equation familiar to enzymologists.

When plotting the specific growth rate (*µ*) against the residual growth-limiting substrate concentration in minimal medium (Fig. 2b), it can be observed that, under saturation conditions (area in grey), *µ* is equivalent to the exponential phase of growth: essentially *µ*_max_, and the initial part of the curve, is the result of substrate depletion, such that growth at *µ*_max_ is not supported. These simple plots can be used to estimate the affinity of an organism for a specific substrate. If an organism has a high affinity for a substrate (a low *K_s_* value), the growth rate will not be affected until the substrate concentration is low. Conversely, if an organism has a low affinity for a growth-limiting substrate (a high *K_s_* value) then the residual concentration of the growth-limiting substrate may remain high, even when the growth rate is affected.

## Prolonging the growth phase

The addition of fresh medium to a culture of microorganisms coupled with the continuous removal of medium and biomass is the basis of the chemostat culture. The chemostat consists of a perfectly mixed suspension of biomass, to which medium is fed at a constant rate. The culture is harvested at the same rate, resulting in a constant culture volume [3]. Simultaneously invented by Monod and Novick and Szilard [4], chemostat culture permits control of the growth rate of the organism from zero to the maximum specific growth rate (*µ*_max_). The introduction of fresh medium at a constant rate and the maintenance of the culture vessel at a constant volume will result in a homogenous steady-state culture. Biomass formation in this system is restricted by a single growth-limiting substrate (see equation 9), with all other nutrients remaining in excess.

The flow of medium into the culture vessel is related to the volume of the culture by the so-called dilution rate (*D*), where:

(10) D=F / V

with *F* being the flow rate of medium into the vessel (l h^−1^) and *V* is the volume of the culture (l), with the units for *D* being h^−1^.

Due to the addition of medium to the culture, there is a net change in the concentration of cells over a period of time, such that:

(11) dX/dt=growth−output

which can be expressed as:

(12) dX/dt=μX−DX

In the chemostat, removal of medium and biomass occurs at the same rate as new medium enters the vessel. The culture is in steady state, and the cell concentration remains constant, with d*X*/d*t*=0, such that *µX*=*DX,* and therefore *µ*=*D*.

Thus, in a steady-state chemostat culture, the specific growth rate (*µ*) is directly controlled by the dilution rate (*D*). A corollary of this is that chemostats can only be operated at dilution rates equal to or below the maximum specific growth rate (*µ*_max_), otherwise the culture will wash-out of the vessel when *D*>*µ*_max_. It is generally accepted that three culture volumes are required to pass through the chemostat for steady state to be achieved [4].

Controlling the growth rate of a culture by varying the dilution rate enables the investigator to study a microbial population of cells at constant growth rate in a homogeneous environment. The chemostat has found great utility in the study of bacterial physiology, environmental factors such as pH, nutrients, cellular perturbations, and in evolutionary studies [4]. A great advantage of these approaches is that physiology can be studied at specific growth rates independent of secondary growth effects, and numerous studies have shown the potential to enhance reproducibility in ‘omics-type studies [4].

## Summary

This primer on microbial growth kinetics highlights the value of a growth experiment that is conducted carefully and analysed appropriately. It also serves as a refresher on the insights that can be extracted from the humble growth curve and reminds researchers of the need for growth curves to be constructed as semi-logarithmic plots. It is intended as a prompt to researchers to consider how to share growth parameters and data with other researchers to allow comparisons of strain growth behaviour between laboratories [7]. This ensures that most value can be extracted from published data.

The ease of access to 96-well plate readers and the emergence of high-throughput 96-well-based phenotyping of bacterial strains means that it is easier than ever to conduct large-scale replicate studies of microbial growth. Semi-automated analysis methods make it easy to analyse these data rapidly and provide the parameters craved by other researchers, with dedicated open-source software emerging for analysis (e.g. [8–10]).

The value of considering how and why an organism behaves in a particular manner can be highly informative when attempting to understand a system, to understand a particular behaviour or to elucidate a mutant phenotype. There is still much to be discovered about bacterial growth, the factors that affect the physiology of growing cultures and how this can be used to determine underlying cellular processes [11]. Measuring the growth rate under specific conditions is a simple way to define the physiological state of cells and observe how microbial cells behave. Remember, Jacob, Lwoff and Monod received their 1965 Nobel Prize through their ability to carefully construct bacterial growth curves and observe what was happening to the cells in their cultures, leading to ‘their discoveries concerning genetic control of enzyme and virus synthesis’ (https://www.nobelprize.org/prizes/medicine/1965/summary/).
